# Over-the-scope clip on over-the-scope clip technique for closure of delayed perforation near a previously placed over-the-scope clip

**DOI:** 10.1055/a-2519-3173

**Published:** 2025-01-31

**Authors:** Susumu Banjoya, Yohei Minato, Yuki Kano, Kohei Ono, Ryoju Negishi, Hideyuki Chiba, Ken Ohata

**Affiliations:** 1 Department of Gastrointestinal Endoscopy, NTT Medical Center Tokyo, Tokyo, Japan; 2 Division of Gastroenterology, Itabashi Chuo Medical Center, Tokyo, Japan; 3 Department of Gastroenterology, Omori Red Cross Hospital, Tokyo, Japan

The over-the-scope (OTS) clip is effective for defect closure during endoscopic treatment 1 2 . OTS clip closure has been reported to be effective in preventing delayed perforation and bleeding, especially after duodenal endoscopic resection 3 . However, adverse events associated with the OTS clip itself have been reported, including bleeding, perforation, infection, and failure to attach 4 5 .

The patient was a 50-year-old man who underwent endoscopic submucosal dissection (ESD) for an 80 mm, 0-IIa, superficial, nonampullary, epithelial tumor in the descending duodenum. After endoscopic treatment, the ulcer was closed with OTS clips ( Fig. 1 ). Fever and abdominal pain persisted after endoscopic treatment, and contrast-enhanced computed tomography scan of the abdomen on the third day after endoscopic treatment showed free air and abscess formation around the outside of the resection site ( Fig. 2 **a** ). Emergency upper gastrointestinal endoscopy was performed because of delayed perforation. We tried to close the perforation, but the fragile edges of the perforation made it difficult to close with conventional hemostatic clips. Then, we pulled the edge of the perforation site and the tissue grasped by the previous OTS clip together, and secured them with a new OTS clip ( Fig. 3 , Video 1 ).

**Fig. 1:**
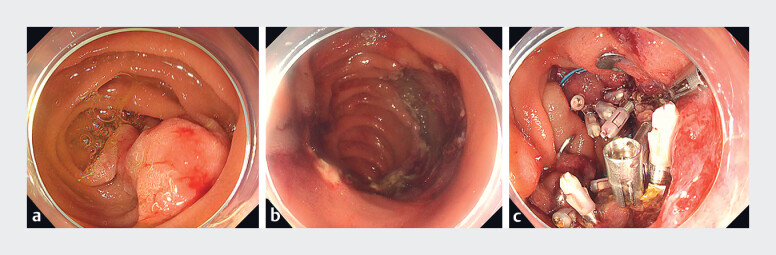
Endoscopic submucosal dissection (ESD) and defect closure. **a** The 80 mm, 0-IIa, superficial, nonampullary, epithelial tumor in the descending duodenum. **b** After ESD. **c** After suture of the ulcer base with an over-the-scope clip and standard clips.

**Fig. 2:**
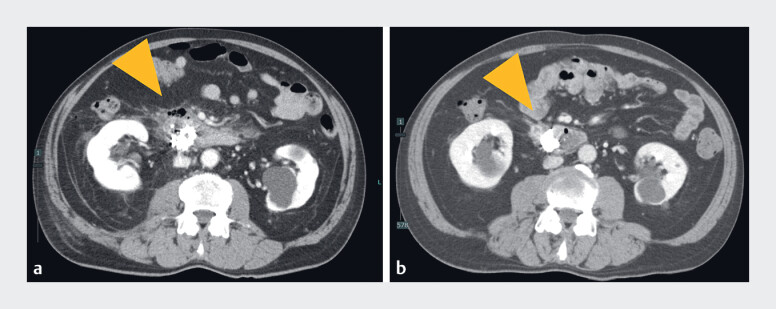
Contrast-enhanced abdominal computed tomography after endoscopic submucosal dissection (ESD) showing abscess (arrowhead). **a** 3 days post-ESD. **b** Before discharge (37 days post-ESD).

**Fig. 3:**
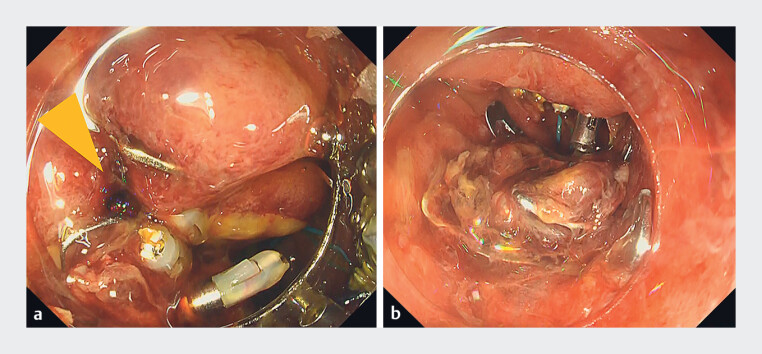
Emergency endoscopy. **a** Perforation was observed near the over-the-scope (OTS) clip used for ulcer closure after endoscopic submucosal dissection (arrowhead). **b** The mucosa around the perforation and the mucosa that the OTS clip had grasped were sutured with a new OTS clip. Closure of the perforation was achieved by the new OTS clip over the first OTS clip.

Over-the-scope (OTS) clip on OTS clip technique. The mucosa grasped by the OTS clip was suctioned into the hood together with the OTS clip itself, and a new OTS clip was placed.Video 1

Endoscopy 14 days after ESD confirmed closure of the perforation ( Fig. 4 ). Endoscopy 37 days after closure of the perforation confirmed maintenance of the closure and revealed that the abscess had disappeared ( Fig. 2 **b** ); the patient was then discharged. Pathological findings showed curative resection with adenocarcinoma, pTis (depth M), Ly0, V0, pVM0, pHM0 ( Fig. 5 ).

**Fig. 4:**
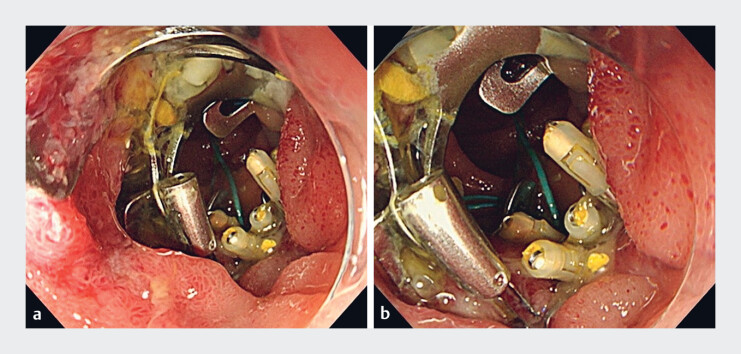
Endoscopy 14 days after endoscopic submucosal dissection confirmed closure of the perforation.

**Fig. 5:**
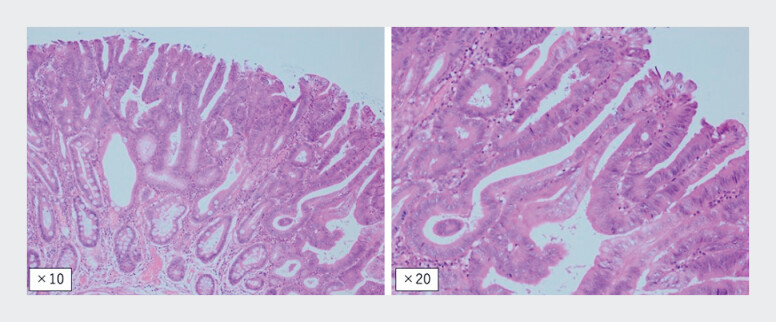
Pathology. The components of the highly differentiated ductal adenocarcinoma were distributed in the range of 10 × 8 mm and 12 × 7 mm. The carcinoma was confined to the mucosa with no invasion into the submucosa. Vascular invasion was negative. The resection margins were judged to be negative, both horizontally and vertically.

OTS clips have a strong grasping force but are difficult to remove once implanted. Using strong grasping force, we tried to pull together the edge of the perforation site and the tissue grasped by the previous OTS clip. Finally, the perforation site, including the previous OTS clip, was completely covered by the second OTS clip.

Endoscopy_UCTN_Code_CPL_1AH_2AZ_3AD

## Notes

Endoscopy E-Videos https://eref.thieme.de/e-videos
*E-Videos* is an open access online section of the journal *Endoscopy* , reporting on interesting cases and new techniques in gastroenterological endoscopy. All papers include a high-quality video and are published with a Creative Commons CC-BY license. Endoscopy E-Videos qualify for HINARI discounts and waivers and eligibility is automatically checked during the submission process. We grant 100% waivers to articles whose corresponding authors are based in Group A countries and 50% waivers to those who are based in Group B countries as classified by Research4Life (see: https://www.research4life.org/access/eligibility/ ). This section has its own submission website at https://mc.manuscriptcentral.com/e-videos .
